# Coiled-Coil Antagonism Regulates Activity of Venus Flytrap-Domain-Containing Sensor Kinases of the BvgS Family

**DOI:** 10.1128/mBio.02052-17

**Published:** 2018-02-27

**Authors:** Elodie Lesne, Elian Dupré, Marc F. Lensink, Camille Locht, Rudy Antoine, Françoise Jacob-Dubuisson

**Affiliations:** a University of Lille, Lille, France; b CNRS UMR 8204, Lille, France; c Inserm U1019, Lille, France; d CHU Lille, Lille, France; e Centre d’Infection & d’Immunité de Lille, Institut Pasteur de Lille, Lille, France; f University of Lille, CNRS, UMR 8576, UGSF—Unité de Glycobiologie Structurale & Fonctionnelle, Villeneuve d’Ascq, France; Washington University School of Medicine

**Keywords:** *Bordetella pertussis*, BvgS family, coiled coil, sensor kinases, two-component regulatory systems

## Abstract

*Bordetella pertussis* controls the expression of its virulence regulon through the two-component system BvgAS. BvgS is a prototype for a family of multidomain sensor kinases. In BvgS, helical linkers connect periplasmic Venus flytrap (VFT) perception domains to a cytoplasmic Per-Arnt-Sim (PAS) domain and the PAS domain to the dimerization/histidine phosphotransfer (DHp) domain of the kinase. The two linkers can adopt coiled-coil structures but cannot do so simultaneously. The first linker forms a coiled coil in the kinase mode and the second in the phosphatase mode, with the other linker in both cases showing an increase in dynamic behavior. The intervening PAS domain changes its quaternary structure between the two modes. In BvgS homologues without a PAS domain, a helical “X” linker directly connects the VFT and DHp domains. Here, we used BvgS as a platform to characterize regulation in members of the PAS-less subfamily. BvgS chimeras of homologues with natural X linkers displayed various regulation phenotypes. We identified two distinct coiled-coil registers in the N- and C-terminal portions of the X linkers. Stable coil formation in the C-terminal moiety determines the phosphatase mode, similarly to BvgS; in contrast, coil formation in the N-terminal moiety along the other register leads to the kinase mode. Thus, antagonism between two registers in the VFT-DHp linker forms the basis for activity regulation in the absence of the PAS domain. The N and C moieties of the X linker play roles similar to those played by the two independent linkers in sensor kinases with a PAS domain, providing a unified mechanism of regulation for the entire family.

## IMPORTANCE

The whooping cough agent *Bordetella pertussis* uses the BvgAS sensory transduction two-component system to regulate production of its virulence factors. BvgS serves as a model for a large family of multidomain bacterial sensor kinases. *B. pertussis* is virulent when BvgS functions as a kinase and avirulent when it switches to phosphatase activity in response to modulating signals. Understanding the molecular regulation of those proteins might lead to new antibacterial strategies. Here, we show that the linker regions between the perception and the enzymatic domains shift between distinct states of conformation in an alternating manner in response to signals and that their antagonistic changes control sensor kinase activity. These linker regions and mechanistic principles appear to be conserved among BvgS homologues, irrespective of the presence or absence of an intervening domain between the perception and the enzymatic domains. This work has thus uncovered general molecular mechanisms that regulate activity of sensor kinases in the BvgS family.

## INTRODUCTION

*Bordetella pertussis* is the agent of whooping cough, an acute respiratory disease. Colonization of the human respiratory tract requires the production of virulence factors whose expression is coordinately regulated by a sensory transduction two-component system (TCS) called BvgAS. TCSs are widespread in bacteria and involved in regulating various cellular processes, including metabolism, mobility, bacterial development, and virulence. Perception of a specific signal by the sensor kinase leads to autophosphorylation of the kinase domain and transfer of the phosphoryl group to the response regulator (1–3).

BvgA is a classical response regulator and in its phosphorylated form activates the transcription of the virulence genes, thus setting the bacterium in the virulent, Bvg-positive (Bvg^+^) phase. Under standard culture conditions, i.e., at 37°C and without ligand, BvgS is in a kinase mode. In the laboratory, BvgS shifts to a phosphatase mode upon perception of modulators such as MgSO_4_ and nicotinate or derivatives or under conditions of nutrient restriction or low temperature. This sets the bacteria in the avirulent, Bvg^−^ phase (4–7). An intermediate virulent phase, called Bvg^i^ and characterized by the expression of only a subset of virulence factors, is encountered at intermediate concentrations of modulators (8).

BvgS is a dimeric sensor kinase that serves as a model for a large family of sensor kinases harboring periplasmic Venus flytrap (VFT) domains for signal perception (9, 10). Each monomer of BvgS is composed of two periplasmic VFT domains (Pfam SBP_bac_3) in tandem; a transmembrane (TM) segment; a cytoplasmic Per-Arnt-Sim (PAS) domain; and a histidine kinase (HK), comprising a dimerization/histidine phosphotransfer (DHp) domain and a catalytic ATP-binding (CA) domain (10). They are followed by receiver and histidine phosphotransfer (HPt) domains, which form a phosphorelay (11).

**FIG 1:**
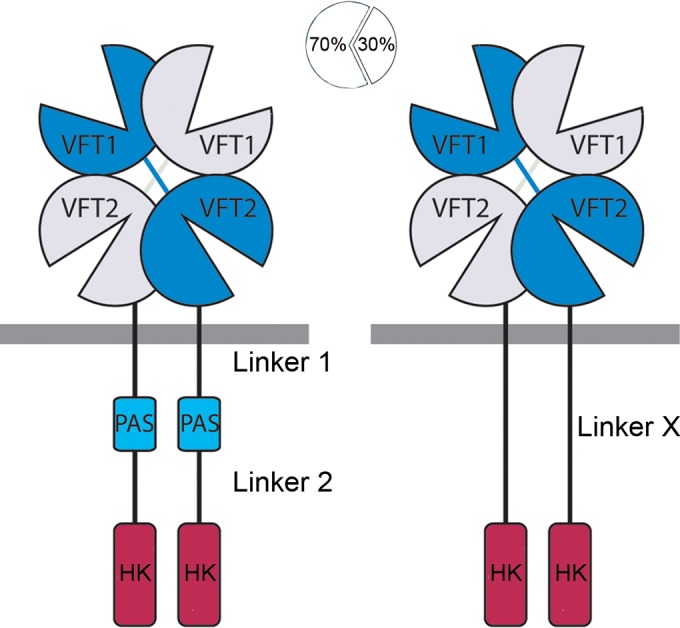
Domain architecture of sensor kinases in the BvgS family. (Left panel) Schematic representation of the BvgS dimer comprising VFT, PAS, and histidine kinase (HK) domains, connected to one another with α-helical linkers 1 and 2. The receiver and HPt domains are omitted for clarity. (Right panel) Approximately 30% of BvgS homologues are devoid of the PAS domain and present an α-helical X linker between the VFT and HK domains. Linkers 1 and 2 are rather highly conserved in size (see Fig. 1 in reference 16 and Fig. 3 in reference 19), while the length of linker X is variable (see Fig. 3 in reference 19). Their sequence conservation is shown in Fig. 9.

Two linkers, called linker 1 and linker 2, connect the VFT domains to the PAS domain and the PAS domain to the DHp domain, respectively (Fig. 1A). Found in many sensory transduction proteins, PAS domains are involved in signal perception and/or in regulatory functions (12–14). We have thus far not found evidence that the PAS domain of BvgS perceives specific chemical or physical signals (15), suggesting that it participates only in signal transduction. The PAS domains of BvgS appear to modify their dimeric interface in response to negative modulators. This change in quaternary structure amplifies small changes of conformation and dynamics of upstream linker 1 (16).

Both linker 1 and linker 2 can form two-helix coiled coils. In canonical coiled coils, the two parallel α helices coil around one another in a left-handed manner. They are characterized by heptads of amino acid residues that form two helical turns, denoted by “abcdefg” letters (17). At the central positions of the coiled coil that form the interhelix interface, the “a” and “d” residues are generally hydrophobic and nonaromatic. The surface-exposed residues are preferentially polar and favor helix formation or mediate inter- and intrahelix interactions. Thus, both hydrophobic effects and ionic interactions stabilize coiled coils (17, 18).

Signal transduction in BvgS proceeds by changes of the conformation and the dynamics of the various segments of the protein. The kinase state is characterized by great dynamics of the membrane-distal VFT1 domains, linker 1 adopting a coiled-coil conformation, the PAS domains forming a dimer, and linker 2 being dynamic (16, 19). This dynamic behavior is compatible with kinase activity, which was shown in model sensor kinases to be linked to dynamic, asymmetrical conformations of the DHp and CA domains in the dimer (20–22). Binding of nicotinate to the VFT2 domains causes BvgS to shift to the phosphatase mode (7, 23). The periplasmic portion of BvgS then becomes less mobile and more compact, which induces a small symmetrical piston movement of the TM helices toward the periplasm (7, 16). This leads to the disruption of the linker 1 coiled coil and the splaying out of the last part of the linker 1 helices, which loosens the PAS domain interface. These changes enable linker 2 to adopt a stable coiled-coil conformation, setting the enzymatic moiety in the phosphatase mode (16, 19).

BvgS serves as a prototype for a family of more than 11,000 VFT-containing sensor kinases, some of which are found in major bacterial pathogens (9, 10). A sizeable proportion (~30%) of BvgS homologues are devoid of a PAS domain (19). In these proteins, α helices predicted to form coiled coils directly link the VFT and DHp domains (Fig. 1). In this work, we investigated the regulation of sensor kinases devoid of a PAS domain. We found that their activity is regulated by an antagonism between coiled coils at the N and C termini of the interdomain linker, very much like linkers 1 and 2 of sensor kinases with a PAS domain.

## RESULTS

### BvgS_Δ65_: an eccentric regulation.

**FIG 2:**
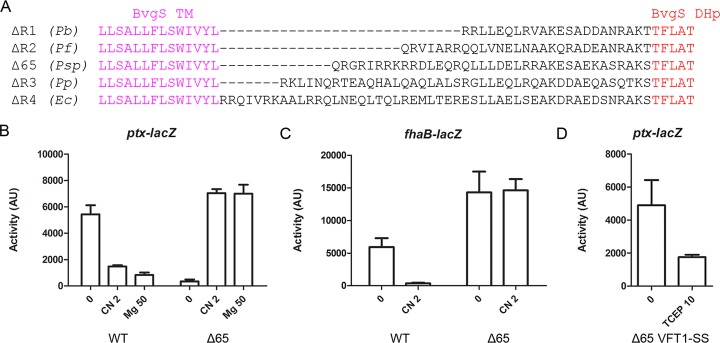
Chimeric BvgS variants with natural X linkers of homologs. (A) Sequences of the variants in which the region between the TM segment and the DHp domain of BvgS was replaced with natural linkers of PAS-less BvgS homologues. The species from which the sequences originated are *Pseudomonas brassicacearum* (accession no. WP_039012684; *Pb*), *Pseudomonas fragi* (accession no. WP_016782378; *Pf*), *Pseudomonas* sp. strain M1 (accession no. WP_009621264; *Psp*), *Pseudomonas putida* (GenBank accession no. AAN69015; *Pp*), and *Enterobacter cloacae* (accession no. WP_050860054; *Ec*). The variants obtained were BvgS_ΔR1_, BvgS_ΔR2_, BvgS_Δ65_, BvgS_ΔR3_, and BvgS_ΔR4_, respectively. (B and C) The *ptx-lacZ* (B) and *fhaB-lacZ* (C) reporters were used to determine the basal activities and the responses to 2 mM chloronicotinate (CN 2) or 50 mM MgSO_4_ (Mg 50) for the BvgS_Δ65_ variant compared to those of wt BvgS (WT). AU, arbitrary units. (D) The *ptx-lacZ* reporter was used to determine the effect of VFT1 domain closing on activity (BvgS_Δ65 VFT1−SS_ variant). Where indicated, the reducing agent Tris(2-carboxyethyl)phosphine hydrochloride (TCEP) was added to the cultures at 10 mM to reduce the S-S bond in VFT1. The means and standard errors of the means are given.

Among the sensor kinases homologous to BvgS and comprising two VFT domains, approximately 30% of the predicted proteins have no PAS domain (19). These proteins harbor a single α-helical linker (here called linker X) directly connecting the TM helices to the DHp domain (Fig. 1). We previously generated four chimeric BvgS variants, BvgS_ΔR1_, BvgS_ΔR2_, BvgS_ΔR3_, and BvgS_ΔR4_, in which linker 1, the PAS domain, and linker 2 of BvgS were replaced with the X linkers of four PAS-less BvgS homologues whose lengths differed by multiples of 7 residues (19) (Fig. 2A). The four chimeras displayed kinase activity at the basal state, and the BvgS_ΔR3_ and BvgS_ΔR4_ chimeras shifted to the phosphatase mode in response to chemical modulation (19). Thus, as BvgS-like regulation can be obtained with linker X sequences, this system may serve as a platform to decipher the rules that govern regulation in the entire family.

Here, we constructed a new chimera called BvgS_Δ65_, whose natural X linker from the *Pseudomonas* sp. strain M1 BvgS homologue is 8 residues longer than that of BvgS_ΔR2_ (Fig. 2A). We characterized the activity of BvgS_Δ65_ using two reporter systems, the *ptx-lacZ* and *fhaB-lacZ* transcriptional fusions (24) positively regulated by BvgAS and downregulated by chemical modulation. Both promoters are activated at high concentrations of phosphorylated BvgA, but only the *fhaB* promoter can be transactivated at low concentrations that correspond to Bvg^i^ conditions (25). At the basal state (i.e., under standard growth conditions), BvgS_Δ65_ exhibited hardly any activity using the *ptx-lacZ* reporter and high β-galactoside (β-Gal) activity levels with *fhaB-lacZ*, indicating that the recombinant strain is in an intermediate, Bvg^i^ phase (Fig. 2B and C). Addition of chloronicotinate, a potent modulator, to the bacterial cultures caused a shift to β-Gal activity levels similar to those of wild-type (wt) BvgS under standard conditions (i.e., in the kinase mode) for the *ptx-lacZ* reporter (Fig. 2B). No change of *fhaB-lacZ* activity was detected under modulating conditions, most likely because the maximum level of *fhaB* transcription was achieved at the basal state (Fig. 2C). Thus, BvgS_Δ65_ regulates *ptx* expression in a manner opposite that seen with wt BvgS, as it shifts to a high-kinase mode of activity upon modulation. However, it is in a low-kinase (rather than phosphatase) mode under basal conditions.

Addition of MgSO_4_, another chemical modulator (4, 26), caused the same increase of *ptx-lacZ* activity as chloronicotinate (Fig. 2B). We also tested the closed membrane-distal VFT1 domain of BvgS, as VFT1 may represent the natural ligand-binding sites *in vivo* (10). Disulfide (S-S) bond-mediated closing of VFT1 to mimic a liganded form shifts wt BvgS to phosphatase, similarly to chemical modulation (10). Introduction of the same S-S bond in BvgS_Δ65_ restored kinase activity at the basal state with the *ptx-lacZ* reporter, and reduction of the S-S bond (i.e., allowing reopening of VFT1) by the use of Tris(2-carboxyethyl)phosphine hydrochloride (TCEP) markedly reduced the kinase activity (Fig. 2D). Thus, regulation of BvgS_Δ65_ is globally inverted relative to that of BvgS. Taken together, our data suggest that the extra residue in linker X of BvgS_Δ65_ relative to the heptad periodicity of the BvgS_ΔR1_ to BvgS_ΔR4_ chimeras displaces the conformational equilibrium away from the kinase mode under basal conditions. Chemical modulation or VFT1 closing shifts BvgS_Δ65_ back to the kinase mode.

### Two coiled-coil registers in linker X of BvgS_Δ65_.

**FIG 3:**
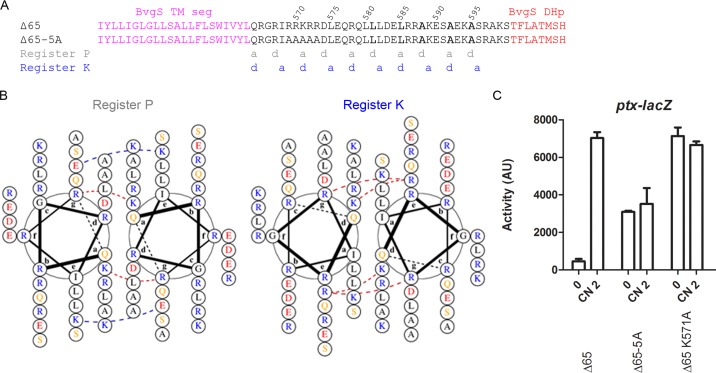
Organization of the X linker of BvgS_Δ65_. (A) Amino acid sequences of the X linkers of BvgS_Δ65_ and the 5-Ala variant. The “a” and “d” positions of the two coiled-coil registers (P and K) are indicated below the sequence. The central “LLAAA” motif is shown in bold letters. (B) Representation of the X linker coiled coils of BvgS_Δ65_ along registers P and K using helical wheel diagrams prepared with DrawCoil 1.0 (http://www.grigoryanlab.org/drawcoil/). Hydrophobic residues are in black, polar residues in yellow, negatively charged residues in red, and positively charged residues in blue. Interfacial electrostatic bridges and core-to-interface electrostatic bridges are indicated with dashed lines, blue for favorable ionic interactions and red for unfavorable interactions. (C) The *ptx-lacZ* reporter system was used to determine the activities of the BvgS_Δ65_ variants. The means and standard errors of the means are given.

Predictions of coiled coils in linker X of BvgS_Δ65_ indicated two distinct registers (Fig. 3A). One of them (in gray letters below the sequence in the figure) is best predicted immediately before the DHp domain of the kinase moiety. The “a” and “d” interfacial positions of that register harbor the Leu and Ala residues of the coiled-coil motif “LxxxLxxAxxxAxxA” (here denoted "LLAAA") found in the linkers 2 of BvgS and its homologs (19). Increased coiled-coil stability resulting from changing the LLAAA motif to LLLLA in linker 2 of BvgS or in the last portion of linker X of the PAS-less BvgS_ΔR1_ variant leads to the phosphatase mode (19). Therefore, the coiled-coil register corresponding to the LLAAA motif is denoted here "register P," for the register inducing the phosphatase mode.

In BvgS_Δ65_, another coiled-coil register (in blue letters in Fig. 3A) is predicted in the first part of linker X, but it appears to be less favorable in the last portion. We called it "register K," for the register inducing the kinase mode. Given that the kinase mode for wt BvgS is characterized by linker 2 being dynamic (19), we hypothesized that for BvgS_Δ65_, the modulated state is characterized by coiled-coil formation along register K in the first portion of linker X while keeping the linker region immediately preceding the DHp domain dynamic.

In BvgS_Δ65_, repulsive interhelical interactions are predicted in the first portion of linker X due to the density of positively charged residues (Fig. 3B). Replacing five consecutive positively charged residues (Arg^569^ to Arg^573^) with Ala generated BvgS_Δ65–5A_, which is locked in the kinase mode but with a lower maximum activity than that of modulated BvgS_Δ65_ (Fig. 3A and C). Among those five residues, the central Lys^571^ residue is in the “d” position of register K, which should affect coiled-coil stability in that register (17, 27). Its replacement yielded a variant locked in a mode of high kinase activity (Fig. 3C). Thus, amino acid residues at key coiled-coil positions determine the balance between the kinase and phosphatase modes and their interconversion.

**FIG 4:**
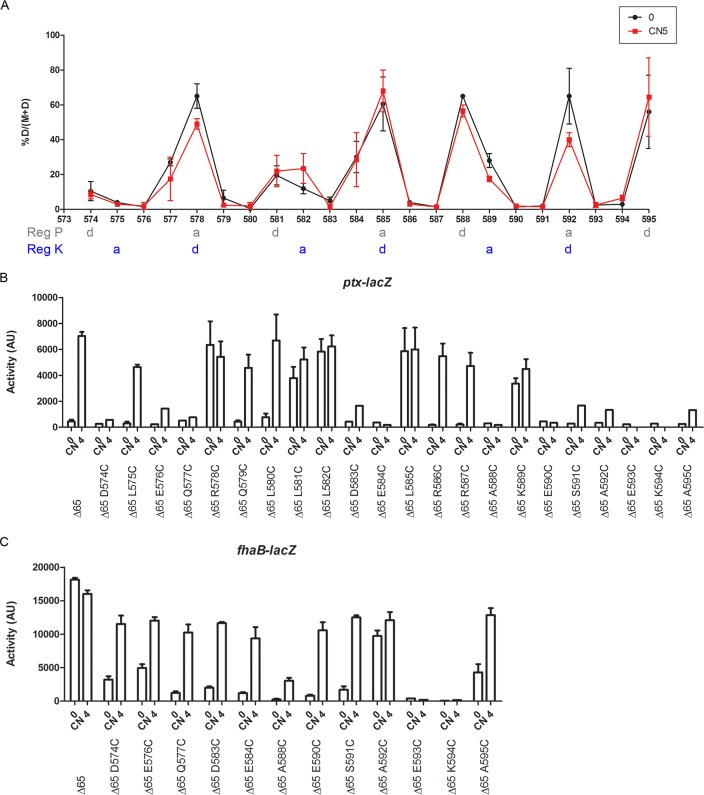
Topology and dynamics of the X linker of BvgS_Δ65_. (A) Cys-scanning analyses of BvgS_Δ65_ were performed under basal conditions (0; black curve) or after the addition of 5 mM chloronicotinate (CN 5; red curve). The proportions of dimers from two independent experiments are graphed, and the means and standard errors of the means are indicated. The “a” and “d” positions of the two coiled-coil registers (Reg P and Reg K) are indicated below amino acid positions. (B and C) The activities of the BvgS variants were determined using the *ptx-lacZ* (B) or *fhaB-lacZ* (C) reporters. The means and standard errors of the means are indicated.

To validate the coiled-coil predictions for linker X of BvgS_Δ65_, cysteine cross-linking-mediated (“Cys-scanning”) analyses were performed after the positively charged region. Residues 574 to 595 were individually replaced by Cys, and an oxidative treatment was performed to favor *in vivo* formation of intradimer S-S bonds (Fig. 4A; see also Fig. S1 in the supplemental material). High proportions of S-S bond-mediated cross-links were observed between residues predicted to adopt interfacial “a” and “d” positions of register P, especially in the portion comprising residues 585 to 595. Coiled-coil formation in register P immediately before the DHp domain is consistent with the close-to-phosphatase (low-kinase) mode of BvgS_Δ65_ under basal conditions. In the region encompassing residues 579 to 584, S-S bond patterns indicated a coiled-coil irregularity that might be the site of interconversion between the two registers. Surprisingly, the addition of a modulator did not modify the proportions of interhelical cross-links formed at most positions. This result might be related to the fact that the protein is not fully in the phosphatase mode under basal conditions and, therefore, that the cross-links represent the superimposition of two conformations, rather than a completely displaced equilibrium. In addition, many Cys substitutions affected BvgS_Δ65_ activity, which might also explain the limited effects of modulation on S-S-mediated cross-linking (Fig. 4B and C). While the regulation trends were at many positions similar to that seen with BvgS_Δ65_, i.e., the kinase activity increased upon modulation, the levels of activity were lower. In addition, several Cys variants appeared to be locked in the kinase mode (positions 578, 581, 582, 585, and 589) or in the phosphatase mode (positions 593 and 594). We checked the production and stability of the latter two in *B. pertussis* membrane extracts to dismiss the possibility that the proteins were proteolyzed *in vivo*. Both were properly produced (Fig. S2A), suggesting that charged residues at positions 593 and 594 are important for BvgS_Δ65_ function. Of note, charged residues were also found at the corresponding positions in variants BvgS_ΔR1_ to BvgS_ΔR4_ (Fig. 2A), as is often the case in the members of that family (see below). The observation that replacements of interfacial and noninterfacial residues affect regulation indicates that coiled-coil stability in each portion of linker X, which is dictated by the nature and the arrangement of both interfacial and surface residues, is critical for function.

10.1128/mBio.02052-17.1 FIG S1  Cys-scanning analyses of linker X of BvgS_Δ65_. The results of a representative Cys-scanning experiment are shown. Cross-linking was performed under basal conditions (-) or after the addition of 5 mM chloronicotinate (CN5) (+). The monomeric and dimeric forms of BvgS_Δ65_^t^ are denoted “M” and “D,” respectively. The “a” to “g” positions of the two-helix coiled-coil heptads are indicated below each substitution. The proportions of dimers, determined as indicated at the top left, are given at the top of each lane. Download FIG S1, TIF file, 0.8 MB. Copyright © 2018 Lesne et al. 2018 Lesne et al. This content is distributed under the terms of the Creative Commons Attribution 4.0 International license.

10.1128/mBio.02052-17.2 FIG S2  Detection of the inactive BvgS variants. Immunoblot analyses of BvgS variants in membrane extracts of *B. pertussis* were performed. The *ΔbvgA* strain is shown as a control to show the abundance of BvgS in an avirulent strain. The loading control was an unidentified *B. pertussis* protein fortuitously recognized by the antibodies (indicated with an asterisk). Note that in several instances spontaneous formation of an S-S bond between monomers resulted in the presence of dimers (denoted “D”). (A) Cys variants of BvgS_Δ65_. (B) Size variants of BvgS_Δ65_. (C) Size variants of BvgS_Δ70A_ and BvgS_Δ70B_. (D) Variant of BvgS_Δ72 LLAAA_ harboring substitutions that destabilize interhelix interactions. Download FIG S2, TIF file, 0.8 MB. Copyright © 2018 Lesne et al. 2018 Lesne et al. This content is distributed under the terms of the Creative Commons Attribution 4.0 International license.

### Effect of linker length on regulation.

**FIG 5:**
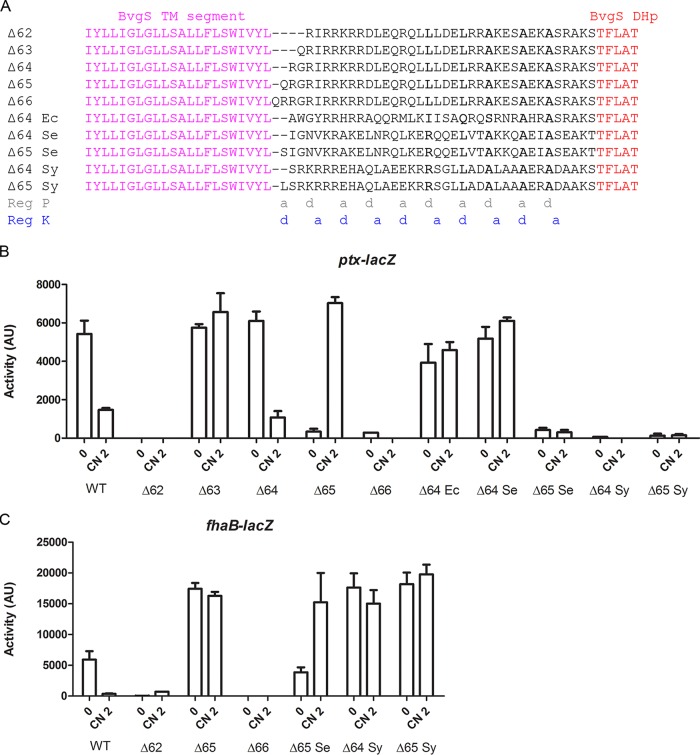
Effect of linker length and composition on activity. (A) Sequences of the X linkers of the size variants of BvgS_Δ65_ (BvgS_Δ62_, BvgS_Δ63_, BvgS_Δ64_, and BvgS_Δ66_) and of other variants are indicated. The species from which the sequences originated were *Enterobacter cancerogenus* (accession no. WP_058610751; Ec), *Selenomonas* sp. strain ND2010 (accession no. WP_081823318; Se), and *Synergistes* sp. strain 3_1_syn1 (accession no. WP_083829553; Sy). The “a” and “d” positions of the two coiled-coil registers (Reg P and Reg K) are indicated below the alignment. (B and C) The activities of the BvgS variants were determined using the *ptx-lacZ* (B) and *fhaB-lacZ* (C) reporters. The means and standard errors of the means are indicated.

To restore the heptad periodicity to linker X of BvgS_Δ65_ as found in chimeras BvgS_ΔR1_ to BvgS_ΔR4_, we deleted one residue immediately after the TM segment, yielding BvgS_Δ64_. We similarly constructed size variants BvgS_Δ62_, BvgS_Δ63_, and BvgS_Δ66_ and tested the effects of those modifications on activity (Fig. 5). BvgS_Δ64_ exhibited regulation similar to that seen with wt BvgS; i.e., it was in the kinase and phosphatase modes of activity under basal conditions and in response to modulation, respectively (Fig. 5B). BvgS_Δ63_ was locked in the kinase mode, while both BvgS_Δ62_ and BvgS_Δ66_ were locked in the phosphatase mode, despite normal BvgS production levels (Fig. 5B and C; see also Fig. S2B). This indicates that the length and the organization of the helices are critical for function of the chimeras. In the context of BvgS, the only functional lengths are those found in BvgS_Δ64_ and BvgS_Δ65_, while the other chimeras are locked in a single state.

### Effect of linker composition on regulation.

We constructed new chimeras harboring natural linkers of the same lengths as that of BvgS_Δ64_ or BvgS_Δ65_ (Fig. 5A). The BvgS_Δ64 Ec_ variant was locked in the kinase mode (Fig. 5B). It notably features a degenerate IQSAA interfacial motif in register P, unlikely to favor coiled-coil formation before the DHp domain. The BvgS_Δ65 Se_ variant exhibited low kinase activity at the basal state, which increased upon modulation, although only with the *fhaB-lacZ* reporter (Fig. 5B and C). It is thus regulated, in an inverted manner, between a phosphatase mode under basal conditions and an intermediate mode of activity under modulating conditions. Another chimera with the same linker size, BvgS_Δ65 Sy_, was locked in an intermediate mode of activity (Fig. 5B and C). The X linkers of BvgS_Δ65 Sy_ and BvgS_Δ65 Se_ harbored interfacial motif RLAAA in register P before the DHp domain (Fig. 5A).

For BvgS_Δ65 Se_ and BvgS_Δ65 Sy_, we deleted one residue to generate variants with linker lengths similar to that seen in BvgS_Δ64_. This deletion locked BvgS_Δ64 Se_ in kinase mode, but BvgS_Δ64 Sy_ remained in the same intermediate state as BvgS_Δ65 Sy_ (Fig. 5B and C). Thus, not all linker X sequences from homologues function properly when inserted between the TM segment and the DHp domain of BvgS. A notable difference between BvgS_Δ65_ and the BvgS_Δ65 Se_ and BvgS_Δ65 Sy_ variants is that the α helices of the former might be stabilized by a number of potential intrahelix ionic bonds between i and i+3 residues or between i and i+4 residues at positions “b,” “c,” and “f” of the coil (28, 29) (Fig. S3). In contrast, the X linkers of the other two chimeras have fewer predicted intrahelix interactions.

10.1128/mBio.02052-17.3 FIG S3  Intra- and interhelical ionic interactions for BvgS_Δ65_ and two other chimeras with X linkers of the same length. The X linkers are represented in the two registers by helical wheel diagrams prepared with DrawCoil 1.0. Hydrophobic residues are in black, polar uncharged residues in yellow, negatively charged residues in red, and positively charged residues in blue. Interfacial and core-to-interface electrostatic bridges (interhelix interactions) are indicated with dashed lines, blue for favorable ionic interactions and red for unfavorable interactions. Potential intrahelix ionic interactions between i and i+3 or i and i+4 residues adopting “b,” “c,” or “f” coiled-coil positions are indicated on one monomer, using black dotted lines. Download FIG S3, TIF file, 1.5 MB. Copyright © 2018 Lesne et al. 2018 Lesne et al. This content is distributed under the terms of the Creative Commons Attribution 4.0 International license.

### Search for functional variants with longer linkers.

**FIG 6:**
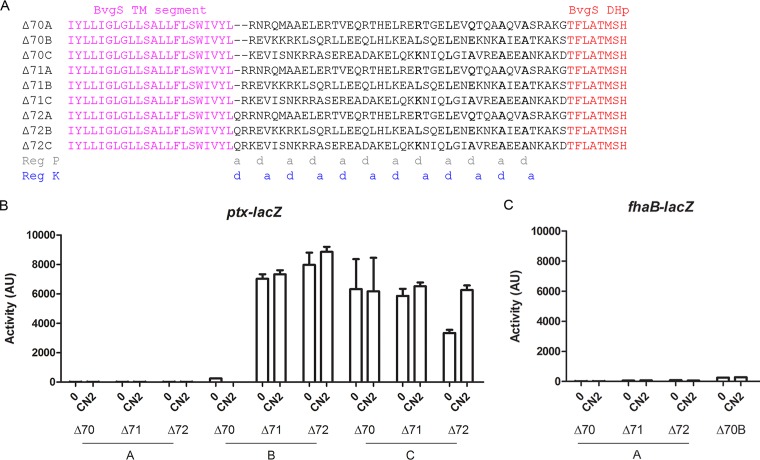
Activity of chimeras with longer linkers. (A) Sequences of the X linkers of chimeric variants. The species from which the sequences originated were *Treponema primitia* (accession no. WP_015709136; Δ70A), *Citrobacter* sp. strain MGH104 (accession no. WP_048232642; Δ70B), and *Butyrivibrio* sp. strain VCB2001 (accession no. WP_081669071; Δ70C). The “a” and “d” positions of the two coiled-coil registers (Reg P and Reg K) are indicated below the alignment. (B and C) The activities of the BvgS_chim_ variants were determined using the *ptx-lacZ* (B) and *fhaB-lacZ* (C) reporters. The means and standard errors of the means are indicated.

We constructed chimeras with X linkers longer than that of BvgS_Δ65_ and screened for functional sequences. In addition, we modified their length to obtain the same heptad periodicities as in BvgS_Δ65_ and BvgS_Δ64_ (Fig. 6A).

BvgS_Δ70A_, BvgS_Δ71A_, and BvgS_Δ72A_ were all locked in the phosphatase mode, although they were all produced at levels similar to the BvgS level (Fig. 6B and C; see also Fig. S2C). BvgS_Δ70B_, although produced at normal levels, was also locked in the phosphatase mode, but the longer BvgS_Δ71B_ and BvgS_Δ72B_ variants were locked in the kinase mode (Fig. 6B and C; see also Fig. S2C). Thus, the linker length affects the phenotype, but modulation cannot regulate the activity of any of the three chimeras.

In contrast, BvgS_Δ72C_, with an additional full heptad relative to BvgS_Δ65_, showed moderate kinase activity under basal conditions that increased after addition of the modulator. The shorter counterparts, BvgS_Δ70C_ and BvgS_Δ71C_, displayed kinase-locked phenotypes. The partially inverted regulation of BvgS_Δ72C_ is somewhat similar to that of BvgS_Δ65_. Specific features of this sequence include the presence of several predicted interhelical ionic bonds, in particular, in the first portion of the linker in register K and in an interfacial motif, KLAAA, in register P (Fig. 6A).

### Molecular dynamics simulations.

**FIG 7:**
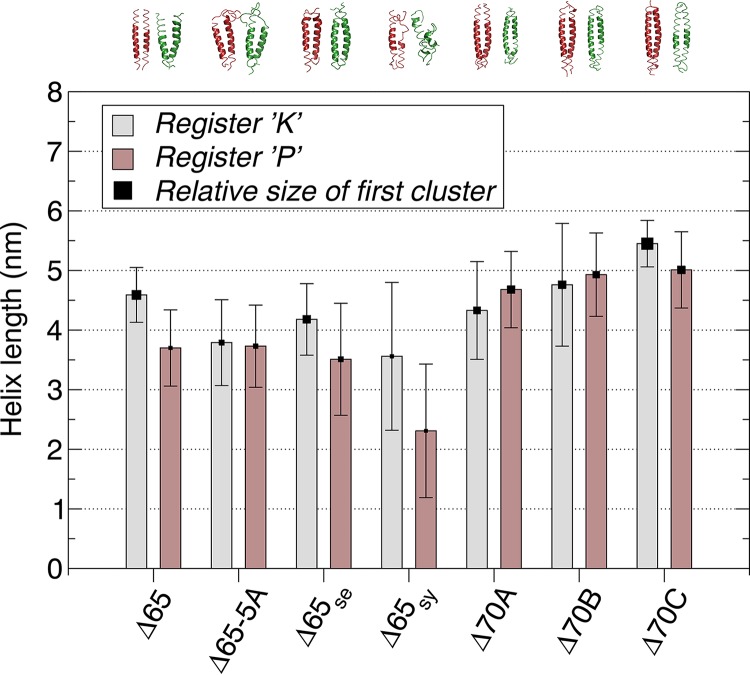
Helix length as calculated from the MD simulations. Averages and standard deviations were calculated for both helices of the coiled coil and for all extracted time frames. Values are shown for each sequence in its assigned registers, namely, registers K (gray) and P (brown). The square data points corresponding to the value of the averages are sized proportionally to the size of the primary (i.e., largest) structural cluster, the largest value being 96.9% for BvgS_Δ70C_ in register K and the smallest value being 24.7% for BvgS_Δ65sy_ in register P. The representative (i.e., “center”) structures of these clusters are shown above the graph, colored red and green for registers K and P, respectively.

We generated *in silico* models of the linkers described above along the two registers and performed molecular dynamics (MD) simulations for each. The simulations were analyzed to determine the features of the linkers that are functional or partially functional in the context of our BvgS platform, i.e., that cause shifts of activity upon modulation. For the BvgS_Δ65_, BvgS_Δ65 Se_, and BvgS_Δ70C_ linker sequences, the levels of helical stability notably differed between the two sets of simulations (Fig. 7). Thus, the α helices along register K remained mostly stable over time, while the linkers tended to form P_i_ helices in register P, mostly at their N-terminal side. For the phenotypically locked BvgS_65-5A_, BvgS_Δ70A_, and BvgS_Δ70B_ variants, in contrast, the MD simulation profiles were almost identical to one another along both registers. MD simulations of the linker of the BvgS_Δ65 Sy_ variant, locked in the intermediate phase, showed strong disruption of helicity over the entire length of the coiled-coil structure in register P and a somewhat less severe disruption in register K. From these data, it appears that the stability of the α helices that form the X linkers contributes to function. In addition, differential levels of α-helical stability between registers K and P enable the interconversion between two states. In contrast, locked phenotypes appear to correspond to low overall helical stability or to similar helical stabilities in the two registers.

### Surface and core residues determine regulation.

On the basis of all the information gathered on those systems, we selected a chimera to use for sequence modifications aimed at altering its regulation properties in a controlled manner. BvgS_Δ72C_ was chosen because its length matches that of BvgS_Δ65_ but with an additional full heptad. In addition, it appears to be weakly upmodulated by chloronicotinate (Fig. 6B). We thus tested our hypotheses of regulation by modifying its composition in a rational manner.

**FIG 8:**
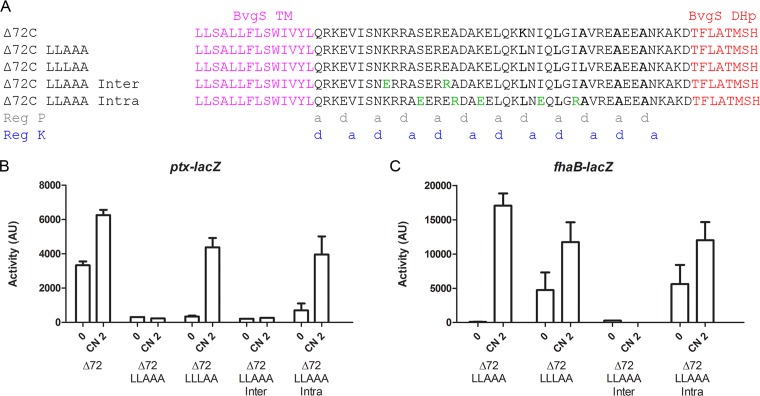
Modifications of the effects of coiled-coil registers P and K on regulation. (A) Sequences of BvgS_Δ72C_ variants where Leu substitutions were progressively introduced to increase coiled-coil stability along register P and of BvgS_Δ72C LLAAA_ variants in which specific amino acids in the "e" and "g" positions or the "b," c," and "f” positions were replaced (denoted in green) to disrupt interhelix interactions or to favor intrahelix interactions. The “a” and “d” positions of the two coiled-coil registers (Reg P and Reg K) are indicated below the alignment. (B and C) The activities of the BvgS_Δ72C_ variants were determined using the *ptx-lacZ* (B) and *fhaB-lacZ* (C) reporters. The means and standard errors of the means are indicated.

To favor the phosphatase mode, we modified the KLAAA motif by consecutively replacing the Lys and first Ala residues by Leu residues in “d” position along register P to increase coiled-coil stability before the DHp domain (Fig. 8A). BvgS_Δ72C LLAAA_ was in the phosphatase mode at the basal state (Fig. 8B and C), and addition of the modulator increased its kinase activity with the *fhaB-lacZ* reporter system (Fig. 8C). Further strengthening the coiled coil by generating the LLLAA motif yielded a bona fide “inverted-regulation” variant, BvgS_Δ72C LLLAA_, whose phenotype paralleled that of BvgS_Δ65_ (Fig. 8B and C). Its linker length corresponded to that of the latter plus one full heptad.

Thus, one can restore regulation by strengthening register P. We tried to obtain the same effect by altering the other register, starting from BvgS_Δ72C LLAAA_. In particular, we modified the inter- or intrahelix interactions in the first segment of its linker X by introducing mutations that exclusively affect residues at noninterfacial positions b, c, e, f, and g of register K. The combined substitutions K_571_E and E_578_R in BvgS_Δ72C LLAAA_, designed to weaken interhelix interactions in register K (Fig. 8A; see also Fig. S4), yielded a variant, BvgS_Δ72C LLAAA Inter_, locked in the phosphatase mode (Fig. 8C). It was produced at normal levels (Fig. S2D). Thus, as we predicted, decreasing coiled-coil stability in register K by altering noninterfacial positions favors register P, with the LLAAA motif in the latter register taking precedence.

10.1128/mBio.02052-17.4 FIG S4  Intra- and interhelical ionic interactions for BvgS_Δ72C LLAAA_ variants. A representation of the X linkers of the indicated variants along the two registers is provided in the form of helical wheel diagrams prepared with DrawCoil 1.0. Hydrophobic residues are in black, polar uncharged residues in yellow, negatively charged residues in red, and positively charged residues in blue. The substitutions are highlighted in yellow. Ionic interactions with interfacial electrostatic bridges and core-to-interface electrostatic bridges (interhelix interactions) are indicated with dashed lines, blue for favorable ionic interactions and red for unfavorable interactions. In BvgS_Δ72C Inter_, three putative favorable interhelix interactions were replaced by unfavorable interactions to weaken coiled-coil formation along the K register. Potential intrahelical ionic interactions between i and i+3 or i and i+4 residues adopting “b,” “c,” or “f” coiled-coil positions are indicated on one monomer in each case, using black dotted lines. Additional intrahelix interactions in BvgS_Δ72C Intra_ compared to BvgS_Δ72C LLAAA_ are indicated with green dotted lines. The substitutions introduced in BvgS_Δ72C Intra_ were aimed at adding putative intrahelix interactions to stabilize the α helices and thus to facilitate the interconversion. Download FIG S4, TIF file, 2.3 MB. Copyright © 2018 Lesne et al. 2018 Lesne et al. This content is distributed under the terms of the Creative Commons Attribution 4.0 International license.

The substitutions S_575_E, A_579_R, K_582_E, I_589_E, and I_593_R were combined to strengthen the α helices by increasing the number of potential intrahelix ionic interactions (Fig. 8A; see also Fig. S4). The resulting BvgS_Δ72C LLAAA Intra_ variant had low kinase activity at the basal state with the *ptx-lacZ* reporter that markedly increased upon modulation (Fig. 8B). The *fhaB-lacZ* reporter system indicated that this variant was in the Bvg^i^ phase at the basal state (Fig. 8C). By increasing helix stability, one could thus obtain the same regulation phenotype as would be obtained by strengthening the coiled coil in register P. This demonstrates that surface-exposed, noninterfacial residues of linker X also contribute to function by determining the intrinsic stability of the helices.

### Conserved elements involved in sensor kinase regulation.

Last, we analyzed a large set of linker sequences encompassing the variety found in the BvgS family to determine whether our findings could be generalized. More than 8,000 sequences of two-component sensor kinases with two VFT domains were collected, including 2,479 proteins without a PAS domain and 5,009 containing a single PAS domain. For the PAS-less proteins, 740 unique sequences of linker X were retrieved and clustered at 80% sequence identity. The first 30 clusters, which are the most highly populated and comprise 393 sequences, were further analyzed. Clusters were grouped depending on heptad composition, yielding four “superclusters” with 214, 53, 39, and 31 sequences, respectively. The sizes of the X linkers range from two to six heptads in this set. Occurrences of longer linkers (up to 13 heptads) in the initial set of sequences were very rare. Linker X of BvgS_Δ65_ belonged to the largest supercluster.

**FIG 9:**
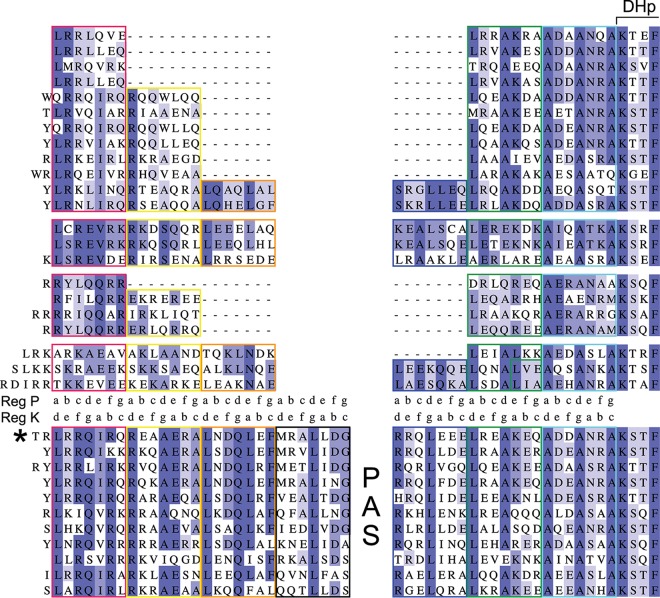
Heptad composition of the linkers in the BvgS family. The sequence alignments of the region between the predicted TM segment and the DHp domain of sensor kinases of the BvgS family are displayed. Each line represents the consensus sequence for one cluster, with the heptads boxed in colors. The first four blocks of sequences (4 superclusters) represent 43% (322 of 740 sequences) of the X linkers of PAS-less proteins. The last block (11 clusters) represents 66.5% (510 of 767 sequences) of linkers 1 and 2 of sensor kinases with one PAS domain. The two registers (registers K and P) are shown in two lines between the two groups of proteins. A large gap, which corresponds to the PAS domain in the second group, was inserted between the first three and the last three heptads of the PAS-less proteins. Note that the fourth heptad (boxed in black) of the proteins with a PAS domain has no corresponding heptad in the PAS-less proteins. BvgS belongs to the sequence cluster indicated by an asterisk.

Sequence alignments showed a high level of conservation in the two heptads that precede the DHp domain (Fig. 9). The LLAAA motif was present in all superclusters, with the exception that the first Leu was not found in the linker sequences harboring only three or four heptads. In addition, a few charged or polar residues were relatively well conserved at non-“a” and non-“d” positions in the last heptads. The conserved features are most likely important for the control of the enzymatic moiety that follows immediately.

In the first heptads of the X linkers, a high density of charged residues was generally conserved, as were a few residues at both interfacial and noninterfacial positions of register K. In particular, a positively charged residue (K^571^ in BvgS_Δ65_ and R^570^ in wt BvgS) shown to be critical for regulation was very often found at the “d” position in the second heptad of the X linkers composed of at least 4 heptads. Overall, conserved features of linker X in the family support our model that its composition generates the coiled-coil antagonism that regulates the output activity of the system.

As for the PAS domain-containing sensor kinases, most harbored four heptads in linker 1 and three in linker 2 (Fig. 9). The sequences of the linkers 2 aligned quite well with those of the last three heptads of the X linkers, except for very short linker sequences (19). Similarly, the sequences of the first three heptads of the linkers 1 aligned relatively well with those of the long X linkers. In contrast, the fourth heptads of the linkers 1 had conserved sequences with no corresponding heptad in the X linkers. This last heptad is most likely necessary to control the PAS domain.

## DISCUSSION

BvgS serves as the prototype for a family of TCS sensor kinases with VFT domains. In BvgS, a cytoplasmic PAS domain flanked by two helical linkers connects the VFT and DHp domains. In a sizeable fraction of BvgS homologues, however, the PAS domain is replaced with a single helical linker between the periplasmic and cytoplasmic parts. In previous work, we identified rules for BvgS regulation (16, 19). Briefly, under basal conditions, linker 1 forms a coiled coil whereas linker 2 is dynamic and BvgS is in the kinase mode. Upon modulation, disruption of the linker 1 coiled coil affects the PAS domain interface, which enables linker 2 to adopt a stable coiled-coil conformation leading to the phosphatase mode (16, 19). In this work, we extended our analyses to the subset of homologues devoid of the PAS domain. We found that linker X between the TM and DHp domains harbors two antagonistic coiled-coil registers. Regulation of activity appears to involve coiled-coil interconversion between these two registers. Therefore, function is governed by similar mechanical principles in the entire family, with specific sequence adaptations in each type of sensor kinase.

In linker X, coiled-coil register P, with the characteristic LLAAA motif also found in linker 2 of PAS-containing family members, has been extensively predicted to be present just before the DHp domain. Coiled-coil formation along that register leads to phosphatase activity, for both PAS-containing and PAS-less sensor kinases (19; this work). In the first moiety of linker X, a moderately stable coiled coil is predicted along register K, with a suboptimal interfacial motif that is compensated for by favorable inter- and intrahelical ionic bonds. Removing interhelical ionic interactions along register K in that region favors the phosphatase mode. Conversely, enhancing coiled-coil formation along register K by strengthening the interface favors the kinase mode, as shown by the kinase-locked phenotypes of the Lys^571^-to-Ala variant in linker X of BvgS_Δ65_ (this work) or the Arg^570^-to-Ala variant in linker 1 of BvgS (19). A charged residue at this position is highly conserved in the family.

We propose that the antagonism between the two coils in the PAS-less BvgS homologues forms the basis for regulation: coiled-coil formation along register K prevents coiled-coil formation along register P and vice versa, and signal perception triggers interconversion. The dynamics of the last portion of linker X is conducive to kinase activity, which has been proposed to require dynamic asymmetry of the DHp helices in model sensor kinases, while the phosphatase state corresponds to a more rigid state (21, 22). By affecting the TM helices, modulation displaces the equilibrium in one direction or the other in a manner dependent on the initial state of the system, which is itself determined by the length of linker X. Such a mechanism of interconversion between two states of coiled coils triggered by external signals has been reported for Nek2, a eukaryotic kinase involved in cell cycle regulation (30). Other bacterial His kinases appear to function in a similar manner (31). Successive domains are connected by two-helix coiled-coil linkers, and a register mismatch between the coiled coil of the linker and that of the preceding or following domain allows conversion between states. Similarly, in the Tar and Tsr chemoreceptors, a “control cable” modulates the intensity of the register mismatch between the TM2 helix and the following HAMP domain (32).

The BvgS_Δ65_ chimera displays inverted regulation; i.e., modulators cause a strong increase of kinase activity. In wt BvgS, modulator binding to the periplasmic domain causes a small piston motion that pulls one residue of linker 1 into the membrane, thus disrupting the first coiled coil (16). In BvgS_Δ65_, modulation is expected to cause a similar motion that shortens the cytoplasmic length of linker X by one residue, in this case leading to the kinase mode. Another way to bring about the kinase mode is to artificially remove one residue from linker X of BvgS_Δ65_, such as in BvgS_Δ64_. Not coincidentally, the length of BvgS_Δ64_ linker X differs by full heptad counts from those of the chimeras of BvgS_ΔR1_ to BvgS_ΔR4_, which are also in kinase mode under basal conditions (19).

The length of linker X is thus a critical determinant of function. Thus, one-residue additions or deletions relative to the functional lengths affect coiled-coil packing, with functional consequences. In addition, the ability to undergo interconversion is determined by the composition of the linker X helices, at both interfacial and noninterfacial coiled-coil positions. Local helical propensities and charges have recently been shown to modulate coil stability in a model system (33). The α helices of BvgS_Δ65_ linker X harbor a number of potential intrahelical ionic bonds that contribute to their stability. Similarly, we were able to recover regulatory activity of BvgS_Δ72C LLAAA_ by increasing helix stability. Taking the data together, responsiveness to modulation thus requires marginally stable coils in the two registers and sufficiently pronounced helical stability to avoid the status of being trapped in a single state. According to this model, phosphatase-locked phenotypes might be explained by a loss of helicity, causing the register P coil in the C-terminal portion to prevail. Intermediate (i.e., low-kinase) locked phenotypes might be due to the accommodation of the two coiled-coil registers by loose α helices (34), resulting in suboptimal conformations.

The X linkers that caused a loss of function in BvgS are most likely functional in their natural sensor kinases. For those sensor kinases, signal perception might trigger conformational changes of the VFT and TM domains that are larger than those seen in BvgS, thus allowing register interconversion and activity regulation. It is also possible that the composition of the cytoplasmic membrane in their host organisms differs from that of *B. pertussis*. Differences in membrane thickness or in lipid composition are likely to affect the length of the TM segment, its motions, and its interactions with the polar phospholipid heads, with a possible impact on regulation.

The major finding of this work is that the N-terminal and C-terminal moieties of linker X functionally correspond to linker 1 and linker 2 of sensor kinases with PAS domains, respectively. The first heptads of the X linkers, similarly to the results seen with linker 1, directly follow the TM segment; they “process” the input signal, which consists of changes of the conformation and dynamics of the VFT domains and the ensuing motions of the TM helices. The C-terminal heptads of the X linkers, as seen with linker 2, are involved in the output of the system, i.e., in the control of enzymatic activity. In sensor kinases without a PAS domain, a balanced antagonism between the upstream and downstream registers of linker X is necessary for interconversion between the two states. Sensor kinases with a PAS domain regulate enzymatic activity with similar antagonism between the coiled coils formed by linkers 1 and 2 (16, 19). The sequences of the fourth heptad of linker 1 of the PAS-containing sensor kinases are relatively well conserved in that subfamily, but there is no corresponding heptad in the PAS-less proteins. It is likely that this heptad directly preceding the PAS domain serves to control the quaternary structure of the latter in response to signals. The PAS domain thus arbitrates between the two linkers, serving as a signal amplifier. Therefore, globally similar principles of mechanical signal transduction are most likely valid for the entire family, with specific adaptations for the presence or the absence of an intervening PAS domain.

## MATERIALS AND METHODS

### Strains, plasmids, and culture conditions.

*B. pertussis* was grown on Bordet-Gengou agar plates for 2 days at 37°C and then cultured in modified stainer Scholte (SS) liquid medium at 37°C under conditions of agitation. To construct BvgS chimeras devoid of the PAS domain, synthetic gene portions (Genecust, Luxembourg) coding for the junction between the TM segment and the DHp domain of BvgS homologues were introduced by BglII-XbaI cassette exchange in pUC19mpla (10) to replace the wt BvgS fragment. Then, the EcoRI-HindIII fragments of the resulting plasmids were transferred into pBBR1-MCS4, yielding pBBRmpla variants as previously described (19). To introduce the two Cys codons used to generate an S-S bond between the VFT1 lobes, the SpeI-BamHI cassette of the pUC19mpla derivative coding for BvgS_Δ65_ was replaced by that extracted from pUC19mosaic containing the Glu^113^Cys and Asn^177^Cys mutations (10). Variants with short deletions or insertions were constructed by site-directed mutagenesis on a pUC19-derived plasmid containing the relevant DNA region, followed by cassette exchange in pUC19mpla and then in pBBRmpla. The recombinant *B. pertussis* strains were obtained by introducing the pBBRmpla variants by conjugation into BPSM_newΔAS_ carrying chromosomal *ptx-lacZ* or *fhaB-lacZ* transcriptional fusions (10). The *bvgS*_*Δ65*_ variants used for Cys-scanning analyses in *Escherichia coli* were constructed by mutagenesis on a pUC19-derived plasmid carrying the sequence coding for the transmembrane segment of BvgS, the linker of *Pseudomonas* sp. strain M1 (GenBank accession no. WP_009621264), and the first part of the BvgS kinase. Cassette exchanges were performed in pPORVPH (19). To check the effect of Cys substitutions on BvgS activity, the pBBRmpla_Δ65_ Cys variants were prepared by cassette exchange as previously described (19).

### Cys-scanning analyses.

The disulfide (S-S)-mediated cysteine cross-linking-mediated (Cys-scanning) analyses were performed in *E. coli* UT5600 carrying pPOR_Δ65_ variants that code for truncated BvgSΔ65 Cys variants (called BvgS_Δ65_^t^), encompassing the VFT, TM, linker X, and HK domains as previously described (19). In this case, however, the variants harbored their native Cys^881^, since this residue was not involved in S-S bond formation. The Cys-scanning analyses were performed at least two times for each position, and the results were reproducible. Full-length BvgSΔ65 Cys variants (called BvgS_Δ65_^fl^) were used to determine the effect of Cys substitution on BvgS activity.

### Molecular dynamics simulations.

Models of the coiled coils were prepared in registers K and P using CCBUILDER (35). The models were processed for molecular dynamics (MD) simulations using the Gromacs suite of programs, version 5.0.4, and the Gromos 43a2 force field (36). C-terminal and N-terminal helix ends were neutralized by capping them with an acetyl group and NH_2_, respectively. The systems were solvated with simple point charge (SPC) water (37) in a rhombic dodecahedron box with a minimum of 1 nm between any atom of the peptides and the box edge and were neutralized by adding the appropriate amount of Na^+^ or Cl^−^ counterions. The systems were then relaxed by performing 2,000 steps of steepest descent energy minimization followed by a 100-ps MD simulation with positional restraints on the heavy atoms, using an integration time step of 2 fs and a neighbor list update performed every 5 steps. For the MD simulations, a temperature bath of 300 K with a coupling constant of 0.1 ps was employed (38), using independent coupling for the coiled coil and the solvent. Pressure was maintained at 1 bar using isotropic pressure coupling with a coupling constant of 1 ps. Bond lengths were constrained to their equilibrium values with LinCS (39). van der Waals interactions were cut off at a distance of 1 nm, and electrostatic interactions were calculated with the particle mesh Ewald method (40). Equations of motion for the water molecules were solved with the SETTLE algorithm (41). The production runs, consisting of 3 independent runs of 500 ns each, used the same settings but employed a time step of 4 fs, enabled by the use of virtual interaction sites (42). Data extraction was done from the concatenated production runs at an interval of 100 ps. Helix length was calculated from the values corresponding to the instantaneous helical rise per residue and averaged over all time frames. Clustering of structures was performed on the basis of the root mean square deviation (RMSD) matrix, using an algorithm described in reference 43 and an RMSD cutoff of 0.3 nm. The center structure of the most highly populated cluster was used for the figure.

### Protein sequence analyses.

hmmsearch output files consisting of all predicted proteins identified from the NCBI nonredundant database (release of September 2017) containing both PF00497 (Bacterial extracellular solute-binding protein, family 3) and PF00512 (His kinase A domain) domains were parsed, considering only hits with an E value of <0.001 for both domains. Using a customized Python script, we selected the sequences containing two VFT domains, which resulted in selection of 8,218 TCS sequences. They were divided in two groups, those without PAS domain (2,479 sequences) and those with a single PAS domain (5,009 sequences), using modified versions of the script mentioned above. TM domain predictions were done for all those sequences, and a few sequences were discarded because of the absence of a clearly predicted TM segment.

Sequences devoid of a PAS domain were then reduced to a fragment starting from the end of the predicted TM segment and extending to the end of the HisKA motif, yielding 740 unique sequences of linker X due to high redundancy of this part of the sequences. Sequences containing a single PAS domain were similarly reduced to the end of the HisKA motif, yielding 767 unique sequences, after discarding atypical sequences in which linker 1 was longer than 34 residues. From this set, the linker 1 sequences were extracted for the alignment from the end of the predicted TM segment to the last residue before the PNP motif that initiates the PAS domain. Similarly, the linker 2 sequences included the residues found after the DIT (Asp-Ile-Thr) motif that terminates the PAS domain and before the beginning of the DHp domain.

The two sets of sequences were clustered using CD-HIT (http://weizhongli-lab.org/cdhit_suite), with an 80% cutoff for the linker X sequences and a 50% cutoff for the linker 1 sequences. The 30 most highly populated clusters of linker X sequences and the 15 most highly populated clusters of linker 1 sequences were further analyzed. Sequences were divided into heptads along register P based on a prior coiled-coil prediction using PCOILS (https://toolkit.tuebingen.mpg.de). Heptad-based manual alignment of different clusters was carried out using Jalview (44), generating four superclusters for the linker X sequences and one for the linker 1 and linker 2 sequences.

### Other methods.

β-Galactosidase assays were performed as described previously (10) with three different clones at different times, and the means and standard errors of the means were determined. The chimeras that gave intermediate-phase phenotypes, i.e., high reporter activity with the *fhaB-lacZ* fusion but no activity with the *ptx-lacZ* fusion, were described as having low kinase activity, while the chimeras yielding no reporter activity with either fusion were described as being in the phosphatase state.

For the detection of inactive BvgS variants in *B. pertussis*, the bacteria were lysed, and the membrane proteins were harvested by ultracentrifugation before immunodetection of BvgS was performed as previously described (19). For the Cys variants, to avoid S-S bond formation during sample handling, 10 mM N-ethyl maleimide was added to the resuspended pellet before lysis. Electrophoresis and immunoblotting were performed as previously described (19).
