# Supplementary figures and images for: Coiled-Coil Antagonism Regulates Activity of Venus Flytrap-Domain-Containing Sensor Kinases of the BvgS Family
Source: mBio. 2018 Feb 27;9(1):e02052-17. doi: 10.1128/mBio.02052-17 (PMC5829827; doi:10.1128/mBio.02052-17)

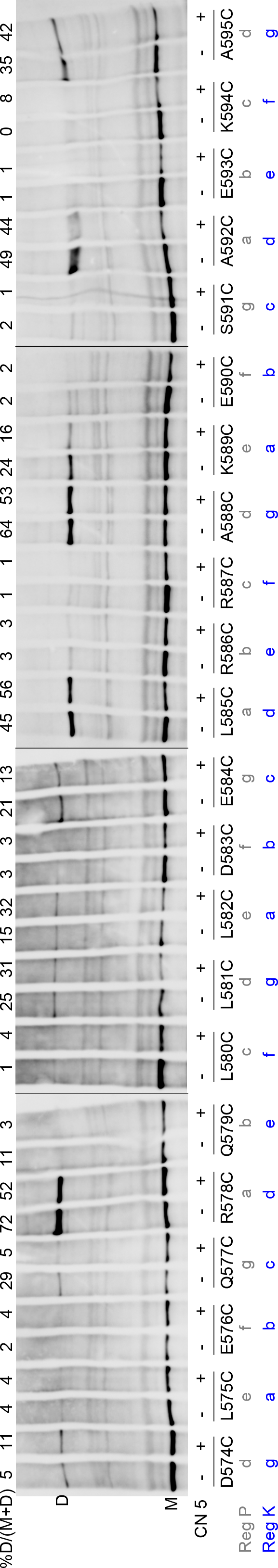

Supplement: FIG S1 [file mbo001183749sf1.tif]

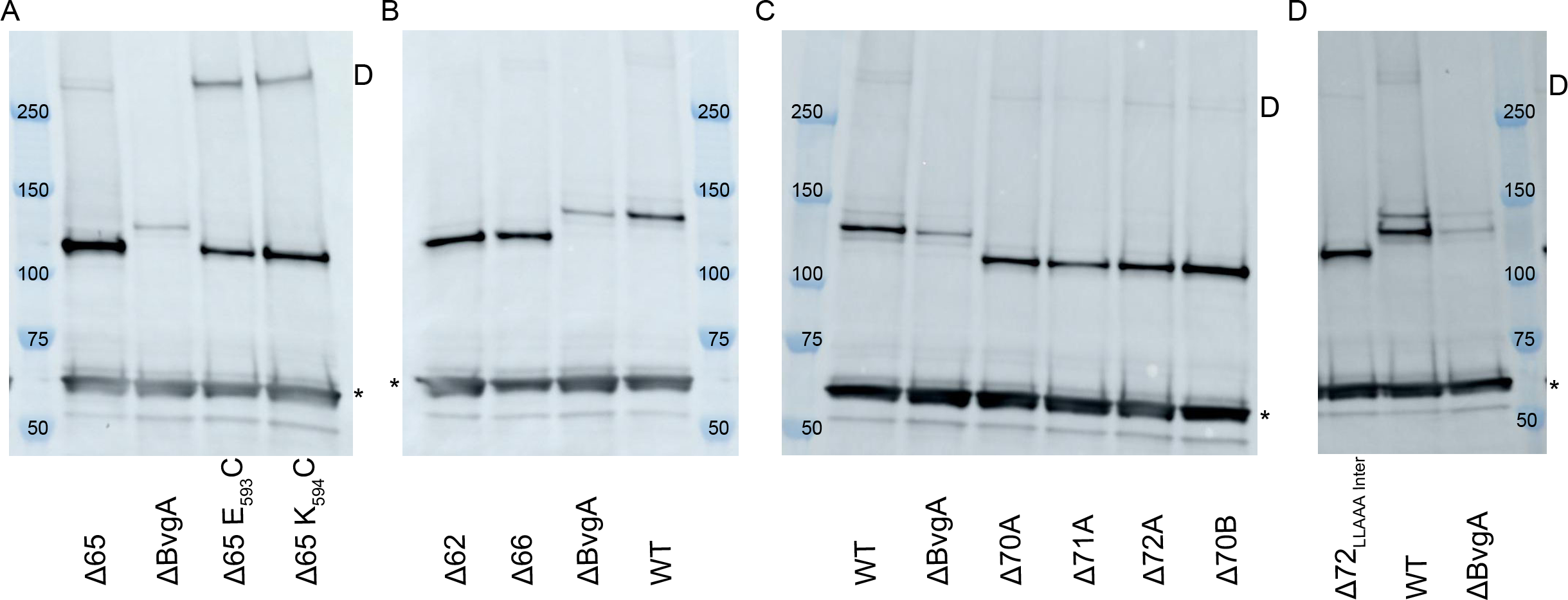

Supplement: FIG S2 [file mbo001183749sf2.tif]

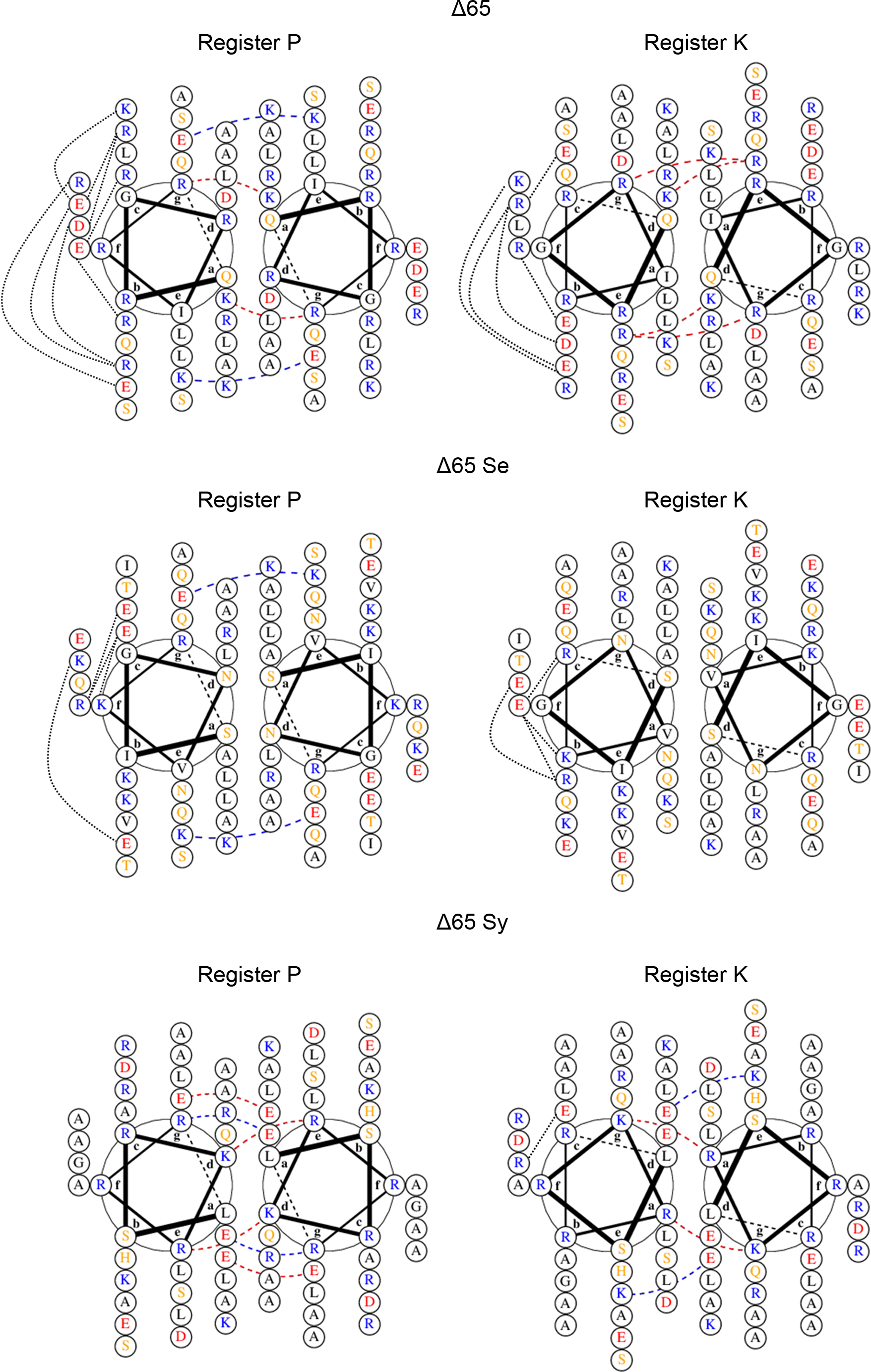

Supplement: FIG S3 [file mbo001183749sf3.tif]

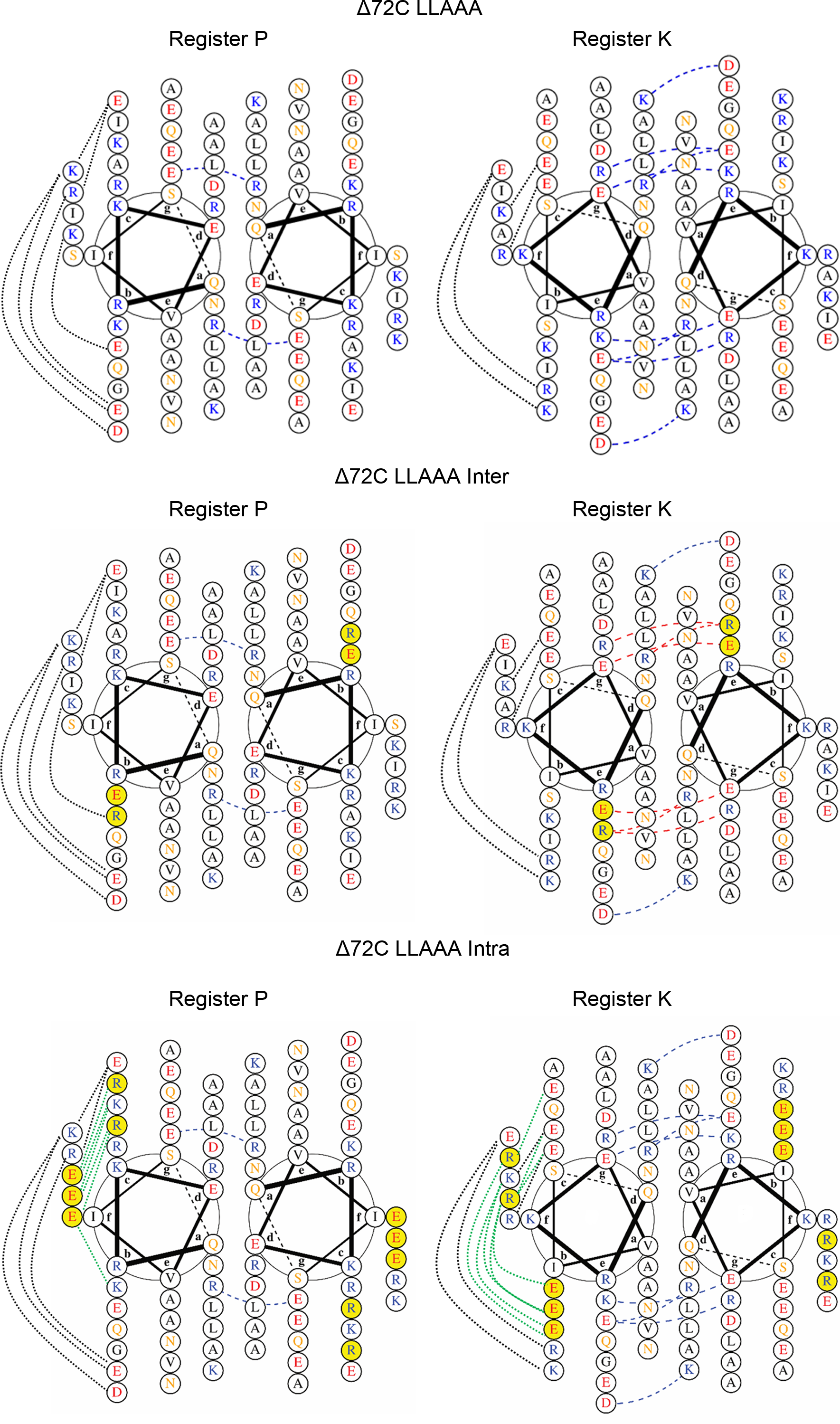

Supplement: FIG S4 [file mbo001183749sf4.tif]
